# Food Chain Transport of Nanoparticles Affects Behaviour and Fat Metabolism in Fish

**DOI:** 10.1371/journal.pone.0032254

**Published:** 2012-02-22

**Authors:** Tommy Cedervall, Lars-Anders Hansson, Mercy Lard, Birgitta Frohm, Sara Linse

**Affiliations:** 1 Chemical Centre, Department of Biochemistry and Structural Biology, Lund University, Lund, Sweden; 2 Department of Biology/Aquatic Ecology, Lund University, Lund, Sweden; RMIT University, Australia

## Abstract

Nano-sized (10^−9^–10^−7^ m) particles offer many technical and biomedical advances over the bulk material. The use of nanoparticles in cosmetics, detergents, food and other commercial products is rapidly increasing despite little knowledge of their effect on organism metabolism. We show here that commercially manufactured polystyrene nanoparticles, transported through an aquatic food chain from algae, through zooplankton to fish, affect lipid metabolism and behaviour of the top consumer. At least three independent metabolic parameters differed between control and test fish: the weight loss, the triglycerides∶cholesterol ratio in blood serum, and the distribution of cholesterol between muscle and liver. Moreover, we demonstrate that nanoparticles bind to apolipoprotein A-I in fish serum in-vitro, thereby restraining them from properly utilising their fat reserves if absorbed through ingestion. In addition to the metabolic effects, we show that consumption of nanoparticle-containing zooplankton affects the feeding behaviour of the fish. The time it took the fish to consume 95% of the food presented to them was more than doubled for nanoparticle-exposed compared to control fish. Since many nano-sized products will, through the sewage system, end up in freshwater and marine habitats, our study provides a potential bioassay for testing new nano-sized material before manufacturing. In conclusion, our study shows that from knowledge of the molecular composition of the protein corona around nanoparticles it is possible to make a testable molecular hypothesis and bioassay of the potential biological risks of a defined nanoparticle at the organism and ecosystem level.

## Introduction

Urgent efforts are needed to make manufactured nanoparticles less reactive if there is any risk that they enter natural environments. Nano-sized particles offer many advantages in biomedical and technical applications [1]–[3] due to unique physical and optical properties related to their small size and large specific surface area. The commercial use of nanoparticles in, for example detergents, cosmetics, food, and dental products, is therefore rapidly growing, leading to a rapidly increasing release of possibly very potent particles into the environment. This specifically raises concerns about nanoparticle effects in freshwater and marine ecosystems since many products containing nanoparticles will end up there through sewage systems.

In a biological fluid, nanoparticles are not pristine, but covered with a protein corona, which mediates the biological effects of nanoparticles [4]–[8]. Previous studies have pointed to a remarkable specificity in the composition of this corona, depending on nanoparticle size and surface chemistry [6]–[7]. The corona is dynamic in nature, and changes over time as the nanoparticle moves from one fluid/compartment to another; the time-dependent evolution of the corona is governed by the relative rate constants, affinities, stoichiometry and concentrations of different proteins [9]. Proteins in the corona may have perturbed structure and aggregation propensity [10]–[13], loss or gain of function [14] or produce an increased inflammatory response [15].

**Figure 1:**
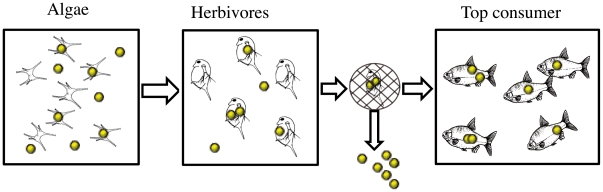
Cartoon illustrating the test food chain with 24 nm polystyrene nanoparticles added at a concentration of 0.01% (w/v) to an algal culture, which after 24 h was filtered and fed to herbivorous zooplankton (algae from 250 ml culture given to 30 adult *Daphnia*). After another 24 h, the zooplankton were gently washed on a net in order to remove remaining or released free nanoparticles before zooplankton were presented to the top consumers of the food chain (fish; 4 individuals per replicate tank). The food chain was restarted every third day and the fish remained the same throughout the study. The control food chain was operated in the same way except that no nanoparticles were added. Each food chain started with 16 fish divided into four tanks. The number of fish in each tank decreased over time due to sacrifice of fish for sampling.

A striking observation is the accumulation of apolipoproteins, particularly apolipoprotein A-I (ApoA-I), in the corona around nanoparticles of several different materials [5]–[7], [16]–[22]. Apolipoproteins are fundamental components of the fat metabolism in most organisms, including humans. Moreover, ApoA-I is a structural and functional protein in HDL (High Density Lipoproptein particles) and forms complexes with phospholipids, cholesterol, and triglycerides. For copolymer particles it is shown that complete HDL bind to the nanoparticles [23]. In most toxicological studies, for example on fish, the studied substances are presented to the organism directly in the surrounding medium. The effects can in those studies be a result of uptake of the substance through the skin or a direct effect on the gills and their function. In a more natural pathway nanoparticles form complexes with substances in nature and are taken up by the fish through food. Therefore we here test the hypothesis that polystyrene nanoparticles are transferred through the aquatic food chain from algae, through zooplankton to fish (Fig. 1) and that the fat metabolism of fish, specifically the mobilization of fat reserves, is affected by nanoparticles.

## Results

**Figure 2:**
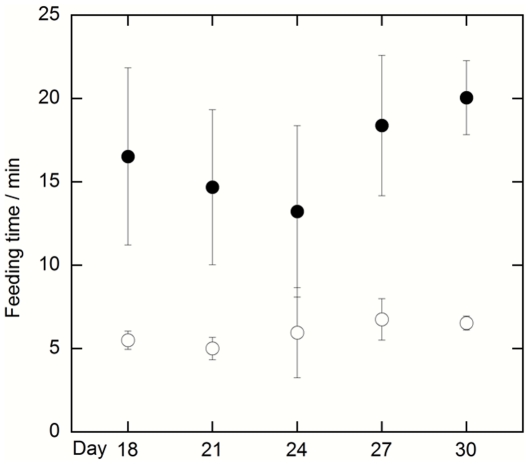
Mean feeding time over four tanks (±standard error of the mean) needed for the top-consumer (fish) to reduce the food (zooplankton) by 95%. Closed symbols denote test fish exposed to nanoparticles and open symbols control fish.

The feeding time (the time it took the fish to eat 95% of the zooplankton added to the tank) was measured at day 18, 21, 24, 27 and 30, and was at all these time points longer for test compared to control fish (Fig. 2). The average feeding time over these five time-points is more than twice as long for fish exposed, compared to not exposed, to nanoparticles (16.6±2.7 and 6.0±0.7 minutes, respectively; F_1,6_ = 33.20; p<0.035; repeated measures ANOVA (analysis of variance)). Ocular observations indicated that test fish were moving more slowly and to a much less extent than control fish and that they did not hunt for zooplankton during the feeding. A striking observation is that test fish let *Daphnia* swim in and out of their mouth without trying to eat them. These data imply a strong behavioural disturbance on the fish after eating food containing nanoparticles.

**Figure 3:**
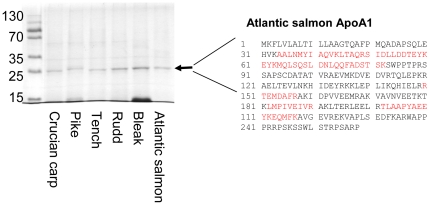
Identification of protein corona on polystyrene nanoparticles in fish serum. Serum from different fish species was mixed with polystyrene nanoparticles. Bound proteins were separated from unbound proteins by centrifugation followed by washing and elution in SDS loading buffer. The eluted proteins were separated according to size by SDS-PAGE. The sequence of Atlantic salmon (*Salomo salar*) apoA-I is shown to the right with peptides identified by mass spectrometry analysis after tryptic digestion of the 25 kDa protein band in red. The identified peptides cover 31.8 percent of the sequence.

Many spherical nanoparticles bind fat-carrying apolipoproteins and lipoproteins that are essential for the fat metabolism. In human plasma several apolipoproteins bind to polystyrene nanoparticles [7] and among them apoA-I. To identify proteins that bind to polystyrene particles in fish serum, we incubated polystyrene nanoparticles with serum collected from several fish species. (Fig. 3). For all fish species investigated, one of the main proteins bound to the nanoparticles migrates as expected for a protein with molecular weight around 25 kDa. The protein band from Atlantic salmon (*Salomo salar*) was cut out and subjected to trypsin proteolysis followed by mass spectrometry. Atlantic salmon was chosen because its genome has been sequenced, and the molecular weights of the tryptic peptides identify the protein as apoA-I (Fig. 3). The sequence identity for apoA-I from different fish species is as low as 40% which makes protein identification difficult in species with unknown amino acid sequence. However, apoA-I from most fish has a molecular weight around 25 kDa [24]–[27] and likely constitutes the nanoparticle bound 25 kDa protein from Crucian carp (*Carassius carassius*) and the other species.

**Figure 4:**
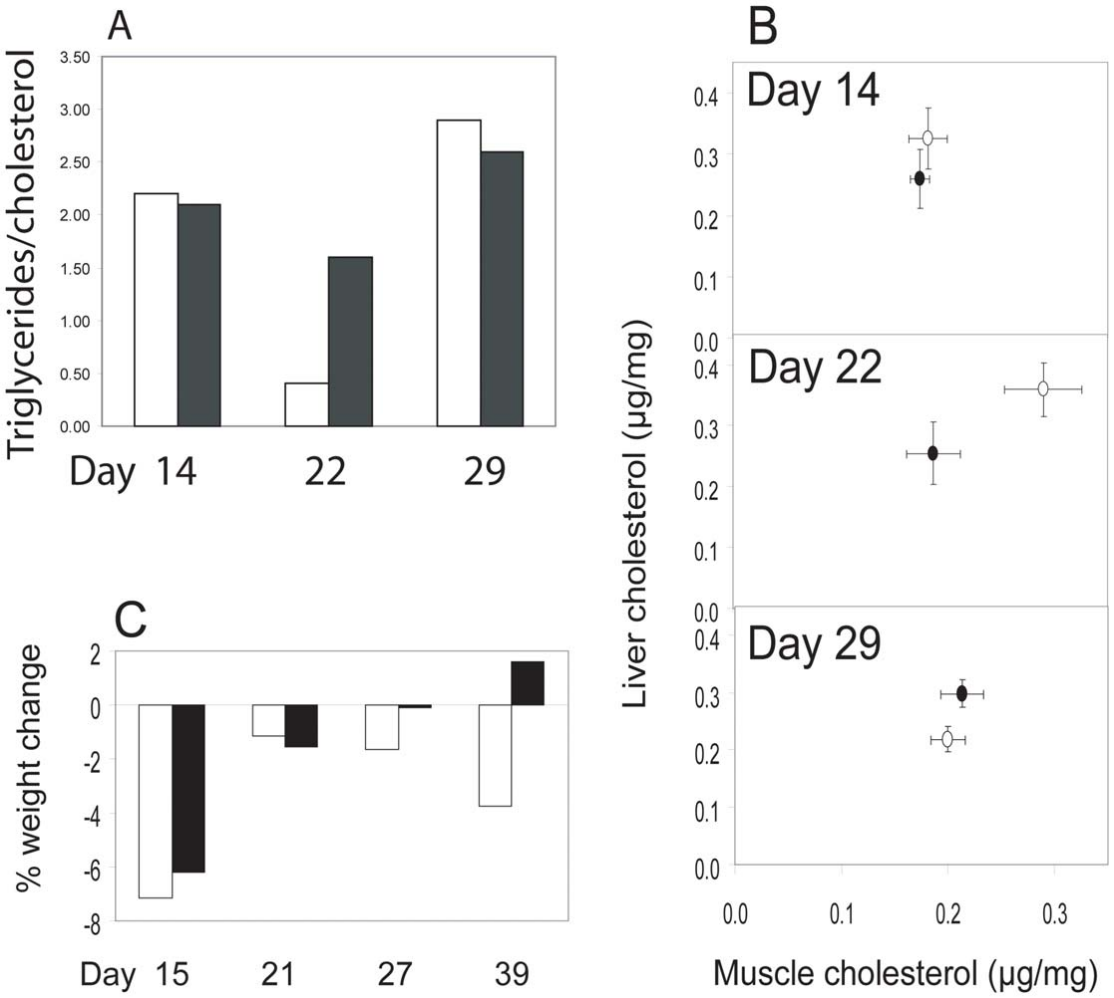
Metabolic changes in fish over time. **A**. The ratio of triglycerides to cholesterol in blood serum of Crucian carp (*Carassius carassius*) exposed (filled bars) and not exposed (white bars) to food containing polystyrene nanoparticles. Each bar shows the average over 4 fishes. **B**. Mean distribution (± average deviation) of cholesterol (µg/mg) in liver (y-axis) and muscle (x-axis) of Crucian carp (*Carassius carassius*) over four fishes per data point. Black symbols denote nanoparticle-exposed and white symbols denote control fish. **C**. The average weight change at each time point relative to the preceding time point is shown (for day 15 relative to day -4). Filled bars refer to the nanoparticle-exposed fish and white bars to the control group. The bars show averages over 16, 12, 8 and 3 control fish, or 16, 12, 8 and 2 test fish, at day 15, 21, 27 and 39, respectively.

ApoA-I and HDL are important in the lipid metabolism. While the data above imply that polystyrene nanoparticles bind Apo AI in serum of our test fish, it is likely that the nanoparticles bind apoA-I and HDL in blood after being taken up through the intestinal wall and travelling to blood and other organs. The nanoparticles might thereby influence the lipid metabolism. As an attempt to register changes in the lipid metabolism we followed the concentrations of triglycerides and phospholipids in blood serum, liver, and muscles throughout the experiment. Two distinct differences were seen between the control and test group. The triglycerides∶cholesterol ratios in blood serum were similar after 14 days (Fig. 4A). After 22 days the ratio for the control fish was very low, whereas only a small decrease was seen in test fish. After 29 days the ratios had increased for both the control and test group. In addition, the distribution of cholesterol among muscle and liver changed during the experiment (Fig. 4B). After 14 days the distribution of cholesterol was the same in control and test fish. However, after 22 days the cholesterol concentrations in the control group were elevated in muscle and liver (Fig. 4B). After 29 days the distribution of cholesterol was again similar.

Throughout the experiment the weight of the fish was measured. A change in weight is a good parameter of the overall metabolic status of the fish. As the fish is fed with a limited amount of zooplankton, a weight loss is expected because the fish is forced to use its energy reserves. A significant weight loss was observed for both the test and control group from the first feeding to the 15^th^ day of the experiment (Fig. 4C). Between day 15 and 21 the weight loss slowed down for both groups. After this, the control group continued to lose weight whereas the test fish actually gained weight at the end of the experiment. A likely explanation is that in the beginning of the experiment both control and test fish used the same energy reserves, resulting in weight loss. Later, from day 15, the control fish continued to use the energy reserves and therefore continued to lose weight. The test fish, however, were inhibited from utilizing the energy reserves due to an accumulation of nanoparticles.

**Figure 5:**
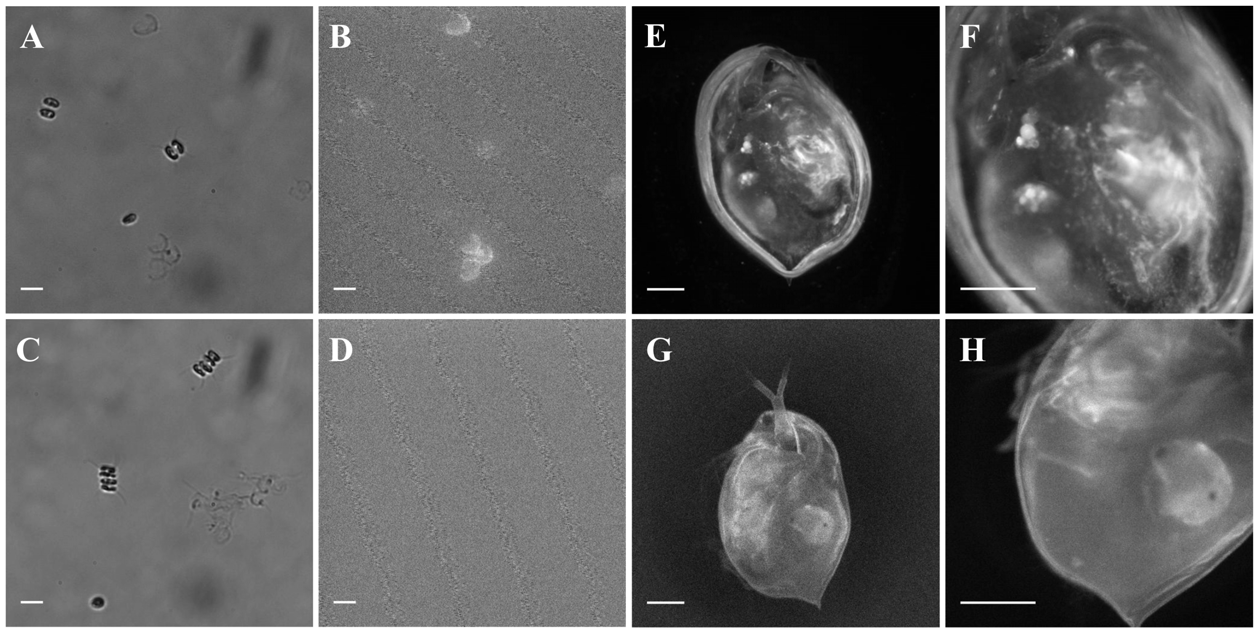
Fluorescence and bright field images of test and control algae and *Daphnia*. **A**. Bright field images of algae taken from test sample, After 24 h incubation with 28 nm fluorescently labeled nanoparticles. **B**. Fluorescence micrograph of algae from A, imaged with Deep Blue filter cube (see Methods), here algal cells are fluorescent due to adsorption of fluorescently labeled nanoparticles. **C**. Bright field images of algae taken from control sample. **D**. Fluorescence micrograph of algae from C, imaged with Deep Blue filter cube, here algal cells are clearly non-fluorescent. Scale bars, A–D: 10 µm. **E**. Fluorescence micrograph of *Daphnia*, taken after 24 hrs incubation with test algae and a light wash through filter. **F**. Close up of E, nanoparticles can be seen in and on the *Daphnia*. **G**. Fluorescence micrograph of control *Daphnia*, taken after 24 hrs incubation with control algae and a light wash through filter. **H**. Close up of G, note some auto-fluorescence in the gut and heart and developing offspring, much of which is distributed evenly. Scale bars, E–H: 500 µm.

Nanoparticle transport through the food chain was studied in a parallel experiment using 28 nm polystyrene nanoparticles with encapsulated fluorescent molecules. After 24 hours test algae show marked fluorescence (Figure 5A,B), whereas no fluorescence was observed from control algae (Figure 5C,D). Control and test algae were given to *Daphnia* that were imaged after another 24 hours. A large number of nanoparticles were clearly visible as distinct fluorescent points in test *Daphnia* (Figure 5E,F), whereas diffuse and much weaker auto-fluorescence was found for both test and control *Daphnia* (Figure 5G,H). When Daphnia were imaged before washing on the net, a significant fraction of fluorescent nanoparticles were observed in the surrounding liquid and these were removed during the washing step. These data imply that nanoparticles are transported through the whole food chain and are delivered to the top consumer, fish, through their food.

## Discussion

Nano-sized material, for example, fullerenes [28] ^24^, single walled carbon nanotubes [29] and titanium dioxide [30], are known to cause biochemical changes in the brain of e.g. fish, which can lead to behavioural changes [29]. Moreover, polystyrene nanoparticles taken up through gills and via the blood are transferred to several organs, including the brain [31]. The fish in our study, which were fed polystyrene nanoparticles through a food chain, moved and hunted more slowly, i.e. showed strong behavioural changes compared to the control fish. In addition, at least three independent metabolic parameters differed between control and test fish: the weight loss, the triglycerides∶cholesterol ratio in blood serum, and the distribution of cholesterol between muscle and liver. This strongly suggests that there is a disturbance of the lipid metabolism as a consequence of nanoparticle intake. Interestingly, changes occurred around 22 days after the nanoparticles intake and starving began, indicating that the nanoparticle-related disturbance of the lipid metabolism is a slow process or that an accumulation of nanoparticles is needed for an effect to be observed. Also interesting, it was the control fish that changed while the test fish showed more stable values. A likely explanation is that the normal adaptation to starvation that seen in the control fish is disturbed or inhibited in the test fish because of the nanoparticles in the system, suggesting that nanoparticles make the fat metabolism less adaptable to starvation.

Our study shows that from knowledge of the molecular composition of the protein corona around nanoparticles it is possible to make a testable molecular hypothesis and bioassay of the potential biological risks of a defined nanoparticle on organism and ecosystem level. Like many other spherical nanoparticles, polystyrene binds apoA-I which may lead to consequences in the lipid metabolism. We show that when polystyrene nanoparticles are transported up the food chain, they have devastating effects on the lipid metabolism of top-consumers, in this case fish. Moreover, the nanoparticles affect the behaviour of the fish thereby having potential effects on ecosystem functioning. It is, to our knowledge, the first time a link between the protein corona and an effect on the metabolism and behaviour of an organism and its function at the ecosystem level has been shown. Hence, in addition to showing that nanoparticles used in everyday products may strongly affect top-consumers both behaviourally and metabolically, we also present a procedure on how to test nanomaterials, such that manufacturing can be optimized in order to avoid future potential environmental and health care disasters.

## Materials and Methods

The study complies with the current laws in Sweden; ethical concerns on care and use of experimental animals were followed under permission (M14-04) from the Malmö/Lund Ethical Committee. Commercially available polystyrene nanoparticles were purchased from Bangs Laboratories Inc. (Fishers, IN, U.S.A.). The diameter was determined by dynamic light scattering (DLS) to be 24 nm in H_2_O. Before the experiments, the nanoparticles were extensively dialysed towards tap water that was used to grow algae, zooplankton and fish in. No aggregation of the particles was observed after dialysis.

We designed a bioassay protocol which as far as possible ensures that the nanoparticles are taken up through food and the intestinal wall. A three-day process was set up for the food chain as follows: day 1) addition of nanoparticles to the algal culture, day 2) feed zooplankton with algae, day 3) feed fishes with zooplankton. This three day process was repeated throughout the 6 week long experiment (Fig. 1). Two parallel food chains were constructed, one with nanoparticles added during algal growth (test) and one with no nanoparticles added (control). The individual steps were performed as follows. On the first day of the process 250 mL of a green algal laboratory culture (*Scenedesmus sp.*, 25 µm in diameter) was distributed to each of eight glass bottles. Four bottles were set aside for the control group. Four bottles were used for the test group and supplemented with 25 mg polystyrene nanoparticles each, yielding a nanoparticle concentration of 0.01% (w/v). The solutions were mixed by shaking for 5 minutes. Algae were then grown together with nanoparticles for 24 hours at 20°C and at a light/dark cycle of 14/10 h (Fig. 1). The 0.01% concentration was chosen to allow a significant number of nanoparticles to be transferred to the fish as a large fraction may remain outside algae and *Daphnia* and be removed during the washing step which follows before presenting *Daphnia* to fish.

Zooplankton (*Daphnia magna*; approximate size 3 mm) were taken from culture and placed in each glass jar (30 adult *Daphnia* per 250 ml jar). On the second day of the process, the filtered algae were added to the respective *Daphnia* jars (test or control) and the jars were incubated at 20°C for 24 hours (Fig. 1). Four control and four test aquaria were filled with 15 liter of water and aerated. Four Crucian carp (*Carassius carassius*) were randomly assigned to each of the aquaria. On the third day of each experimental cycle, *Daphnia* were collected on a 50 µm net and presented to the fish. Fish feeding times were monitored five times (Day 18, 21, 24, 27, and 30), by recording the time it took the fish to eat 95% of the *Daphnia* added to the tank. Differences in feeding time between treatments were tested with Repeated Measures ANOVA.

Four days before the experiment, Day -4, all fish were weighed and measured. Thereafter the fish were weighed before feeding at day 15, 21, 27 and 39. On days 14, 21, 27, and 39 one fish from each tank was removed for blood and tissue sampling. Blood was collected around the gills after decapitation, allowed to coagulate and centrifuged, 2000 rpm, to remove cells and coagulate. The supernatant was centrifuged again at 13000 rpm to remove small aggregates and cell debris and stored in aliquots at −70°C.

Nanoparticle uptake and transfer through the food chain was studied in a parallel experiment using 28 nm diameter polystyrene particles (1% w/v) with encapsulated fluorophore (Duke Scientific Corp., Palo Alto, CA). Green algae (*Scenedesmus sp.*, 25 µm in diameter) were grown in the absence (control) or presence of the fluorescent nanoparticles (test) at a concentration of 0.01% (w/v) for 24 hours at 20°C followed by bright field and fluorescence microscopy. Imaging of samples was performed with an inverted Nikon Eclipse TE2000-U microscope (Nikon Corporation, Tokyo, Japan) using an Andor Ixon EMCCD camera (Andor Technology, Belfast, Northern Ireland), with bright field or epifluorescence illumination with white light or Deep Blue filter cube set (Ex/Em 455/520 nm), respectively. Image acquisition was performed with IQ software (Andor Technology, Belfast, Northern Ireland). The algae were filtered and added to *Daphnia* (test or control) in the same proportions as above and the jars with *Daphnia* were incubated at 20°C for 24 hours. *Daphnia* were collected on a 50 µm net and washed to remove free nanoparticles, followed by bright field and fluorescence microscopy as above.

Fish blood from Crucian carp (*Carassius carassius*), Bleak (*Alburnus alburnus*), Rudd (*Scardinius erythrophthalmus*), Tench (*Tinca tinca*), Pike (*Esox esox*), and Atlantic salmon (*Salmo salar*), was collected and sampled according to the same procedure as for the Crucian carp (see above). From each fish, 100 µl serum was mixed with 1 mg polystyrene particles, 200 nm (to allow pelleting by centrifugation), in PBS and incubated at 23°C for 1 hour. The mixtures were centrifuged, 13000 rpm, for 10 min and the supernatants discarded. The pellets were dispersed in 0.5 ml PBS and the samples centrifuged again. This was repeated once. Proteins bound to the particles in the pellets were desorbed by adding SDS-PAGE loading buffer, and separated on a 12% SDS-PAGE. Proteins migrating at a position corresponding to around 25 kDa were cut out and digested with trypsin.

Trypsin digested peptide extracts were resuspended in 10 µl of 0.1% TFA, and 0.5 µl of each extract was dispensed directly on a MALDI-TOF sample support. The samples were allowed to dry prior to addition of 0.5 µl of matrix solution (5-mg/ml α-cyano-4-hydroxycinnamic acid, 50% acetonitrile, 0.1% TFA, 25 mM citric acid) to each sample. MALDI-TOF mass spectrometry was performed using a 4700 proteomicsanalyzer (Applied Biosystems, Framingham, MA) mass spectrometer in positive reflector mode. For MS and tandem MS (MS-MS) analyses, approximately 1,000 and 2,000 single laser shot spectra were summed up, respectively.

Samples of muscle and liver from Crucian carp were weighed and 1 ml PBS/100 mg tissue was added and the tissue was homogenized. The homogenized tissue was centrifuged, 13000 rpm, 6 min, and the supernatant transferred to new tubes. The absorbance of the supernatant was measured at 280 nm as a control that the procedure resulted in similar levels of homogenization. The triglyceride and cholesterol concentrations were measured in serum, muscle and liver homogenates. Serum Triglyceride Determination Kit, Sigma TRO 100 and Amplex Red Cholesterol Assay Kit, Sigma A12216, were used to determined triglyceride or cholesterol concentrations, respectively, according to the manufacturer's instructions.
